# Student perceptions of scientific writing in pharmacology: Student generation of collaborative rubrics to score a social pharmacology writing project

**DOI:** 10.1002/prp2.1148

**Published:** 2023-10-27

**Authors:** Terri Enslein, Edward Kosack, Hanna N. Wetzel

**Affiliations:** ^1^ College of Nursing Xavier University Cincinnati Ohio USA; ^2^ Williams College of Business, Department of Economics Xavier University Cincinnati Ohio USA; ^3^ Department of Biology Xavier University College of Arts and Sciences Cincinnati Ohio USA

**Keywords:** rubrics, scholarly writing, student perceptions

## Abstract

Scholarly writing is an important skill in all fields of study. Despite a strong focus on writing in many courses, faculty and students have disparate expectations related to scholarly writing. Herein, a classroom exercise is presented in which students were asked to develop a rubric that would be used to evaluate their summative writing assessment. Students were provided with a list of elements that commonly represent good scholarly writing, asked to define what effectively demonstrating these elements looks like, and asked to assign the weight that would be given to each element. The weights given to each element by students were compared to a faculty‐generated, departmental writing rubric. Students assigned significantly higher weights to ideas, and significantly lower weights to sentence fluency. Overall, students favored content over writing mechanics. A random selection of student papers was scored using both the departmental rubric and the student rubric, with about a half‐letter grade difference between the two groups, though the difference was not statistically significant. The outcomes suggest this exercise may be valuable in offering insight into student perceptions of scholarly writing and in furthering student engagement in the writing process.

## Graphical abstract

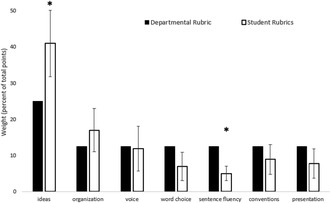

**Abbreviation**
SW: scientific writing

## 1 INTRODUCTION

Effectively training undergraduate pharmacology students in discipline‐specific scientific writing is important for the advancement of the field. Scientific writing (SW) remains a challenge for students in higher education throughout the world, despite varied approaches and incorporations into curricula. Effective communication through scientific writing is an important skill vital to the future success of pharmacologists and, more generally, scientists. SW refers to a clear, concise, and structured piece of professionally formatted writing that is evidence‐based and formal in tone. It must also contribute to the unique voice and thought of the writer, positioning them to contribute to disciplinary knowledge.^1^ SW should also meet spelling and grammar standards and provide an objective perspective.

While there is a plethora of sources, spanning years of academic practice, documenting the struggles that exist with SW in higher education, the same is not true for evidence exploring student views of SW. A review of the literature surrounding both student perspectives and expectations of SW yielded limited results, which are summarized herein. This included a search of Eric, Cinahl, Jstor, and Pubmed using the search terms scholarly writing, perceptions writing, scientific writing, writing expectations, writing practices, higher education writing, student expectations writing, and faculty expectations writing. This speaks to the dearth of available information on the student perspective and supports the need for further research into this area. To adequately understand the issues facing successful SW in higher education, one must position themselves to understand both the student and educator perspectives so that the gap between them can be evaluated for the crafting of meaningful solutions.

Some work has been previously done evaluating both student and faculty perceptions and expectations surrounding scholarly writing in higher education. Many argue that not only do faculty and students not agree on what is meant by scholarly writing, but students also do not value it.^2^, ^3^ Some suggest that students enter into programs having little experience with and confidence in scholarly writing, and many argue that these same students do not understand or value the impact strong writing skills can have on professional practice.^4^, ^5^ What is also evident is that gaps exist in expectations and outcomes between students and instructors. Furthermore, there exists a gap between the students' self‐perceived competence with writing and their actual competence. More concerning, this misalignment of competence and expectation suggests that students often perceive their lowest levels of competence in skills areas that faculty deem the most critical.^6^ This speaks to a lack of clear communication between the instructor and the student.

Ideally, the bridge between faculty and student perceptions is formed by a well‐constructed rubric that both completely and accurately conveys the instructor's expectations and is fully understood by the student. Without this, students tend to rely heavily on previous learning and experience, often disregarding current teaching and guidelines. Such previous learning forms the basis of student understanding to the extent that the student often disregards current guidelines for what is familiar and known ^7^. If that is the case, providing clearer and more explicit guidelines may not help. However, even when the instructor provides a clear rubric, there is often still dissonance between instructor and student expectations.

One innovative approach to addressing this problem is having students play a role in constructing rubrics themselves.^8^ This provides both insight into students' views on scholarly writing and a novel pedagogical approach to SW in the classroom. While themes of misalignment, miscommunication, and lack of confidence and competence are prevalent in the literature, it is difficult to ascertain whether they represent the whole of the issue. Further efforts to explore and discern gaps in SW expectations between students and instructors are needed. Once these nebulous areas have been identified, realistic and targeted solutions can be explored. Herein, we demonstrate how a collaborative rubric exercise, where students assist in writing the rubric that will be used to score their summative writing assessment in an undergraduate pharmacology course, may ameliorate some of these issues with SW. We compare the rubrics generated by the students to standard, faculty‐generated rubrics, highlighting differences between them as opportunities for faculty to engage further with students in the assessment of SW.

## 2 METHODS

### 2.1 Student population

Xavier University is a private, Catholic, liberal arts undergraduate institution. For the 2021–2022 academic year, Xavier had an enrollment of around 4500–5000 undergraduates. For the majors that enroll in Foundations of Pharmacology (Biology, Biomedical Sciences and Biology for Business), out of 538 students 63% identified as White, 3% identified as Asian, 6% identified as more than one race, and 28% identified as belonging to an underrepresented minority in STEM (American Indian or Alaskan Native, Black or African American, Hispanic, or Native Hawaiian or other Pacific Islander). 68% of these students identified as female. Demographic information for the specific class sections involved in this study is unavailable due to a lack of IRB approval to release that information. The class used for this exercise was a 300‐level biology elective titled “Foundations of Pharmacology.” Students were predominantly third‐ and fourth‐year students in the Biology Department. Sections were capped at 30 students. As this course is not required for the major, it is a self‐selected group of students who are interested in the topic and are motivated to learn. This course is a “Writing Flag,” fulfilling the writing‐intensive course requirement for the University Core Curriculum.

### 2.2 Ethical approval and informed consent

All research presented herein was approved by the Xavier University Institutional Review Board. Before being presented with the informed consent statement for review, the instructor of the course left the room. An approved informed consent statement was read to the students by a different faculty member. Students were given paper consent forms to sign, which were collected by the non‐instructor faculty member. Both the statement and the consent form were approved by the Xavier University Institutional Review Board. The informed consent statements were not released until final grades were submitted for the course. Student data were de‐identified before data analysis.

### 2.3 Writing assignment scaffolding

Throughout the semester, students completed a series of scaffolded writing assignments that culminated in a final course paper. For the scaffolded assignments, students were asked weekly to complete a short, graded writing assignment over course content in which they were asked to focus on one specific element of writing: ideas, organization, voice, word choice, sentence fluency, conventions, and presentation^9^ (Table 1). Examples of topics included evaluating black‐box warnings on drug labels, describing different pharmacokinetic parameters, comparing and contrasting different drug mechanisms, and summarizing research findings. These elements were described to the students, and they were provided with the “student friendly” grading criteria from the book by Ruth Culham from which the elements of good writing were adapted. Students were provided with weekly feedback on these aspects of their writing. In addition to the weekly writing assignments, students also read and reviewed two peer‐reviewed pharmacology articles. They critiqued these articles and were asked to think about which elements of writing contributed to producing a good scholarly article.

**TABLE 1:** Elements of scholarly writing adapted from Ruth Culham's “Teach Writing Well: How to assess writing, invigorate instruction and rethink revision (2018)”. Students were introduced to these elements weekly throughout the semester. The table was adapted for the application to college‐level scientific writing.

Writing element	Description
Ideas	Finding a topic, focusing on the topic, developing the topic, using details
Organization	Creating a lead, using sequencing words and transition words, structuring the body of the work, and ending with a sense of resolution (this often means a strong conclusion paragraph in scientific writing)
Voice	Establishing tone, conveying purpose, creating a connection with the audience, taking risks to create voice (for example, asking students to step outside their comfort zone and try new things in their writing)
Word Choice	Applying strong verbs (often this means avoiding the passive voice when possible), selecting striking words and phrases, using words that are specific and accurate, utilizing language effectively
Conventions	Checking spelling, punctuating paraphrasing effectively, capitalizing, correctly, applying grammar and uses
Presentation	Use of word processing, using white space, incorporating text features, formatting of figures, tables, graphs and captions
Sentence Fluency	Capturing smooth and rhythmic flow, crafting well‐built sentences, varying sentence patterns, breaking “rules” to create fluency (for example, using the passive tense or first person when appropriate in scientific writing)

### 2.4 Social pharmacology review writing project

The final writing project for this course was to conduct a literature review, develop a thesis statement, and write a review paper on the interaction of a drug and society. Each student chose their own drug to write about. Students were required to characterize the pharmacokinetics and pharmacodynamics of their chosen drug and defend a thesis statement with peer‐reviewed literature on how a social factor impacted the development or post‐market performance of the drug. For example, one student wrote about off label prescription of semaglutide drugs for weight loss, the demand for which was spurred by social media influencers. Another wrote about the history of psilocybin and how misinformation and stigma may have led to this compound being understudied as an antidepressant. By this point, the course had already surveyed pharmacokinetics, pharmacodynamics, PK/PD interactions, systems pharmacology and drug development.

### 2.5 Collaborative rubric workshop exercise

At the end of the semester, student groups were tasked with developing their own SW rubric to be used for grading of the final course paper. Students were placed in groups at the beginning of the semester, and these groups were retained through the completion of this assignment. Each group made their own rubric, for a total of 5 rubrics per class. For the rubric assignment, each group was again provided with the student‐friendly scoring guide from Culham's book. Group sizes ranged from 2 to 5 students each. All groups started at 4–5 students, but, due to attrition, some groups were smaller by the time the rubric activity occurred, which could impact the results of this study. During a 50‐minute class period, student groups were tasked with creating the rubric that the instructor would use to score each group member's final course paper, worth 15% of the final course grade. While students were in groups for the development of the grading rubrics, each student was responsible for writing their own individual paper. In constructing the grading rubrics, students were asked to define what effectively demonstrating the elements of SW might look like and were asked to decide the weights that would be given to each element (if any) within the rubric. For example, students could choose to be evaluated entirely on ideas and voice with no other writing elements given any weight in the rubric, or they could choose to be evaluated on a mixture of all the elements. The instructor was present during this workshopping class to facilitate discussion and ensure that usable rubrics were generated.

### 2.6 Analysis of rubrics and statistics

Student rubrics were mapped to the elements of writing originally provided for analysis to determine which elements students chose to be evaluated on. If student groups created a category on their rubrics that included parts of multiple elements from the guide written by Ruth Culham (Table 1), the percentage assigned by the group was divided evenly between the relevant elements for purposes of analysis. The standard, faculty‐developed rubric for the assignment from the Biology Department was also used to assess writing, and these departmental criteria were mapped to the selected writing elements for comparison. This rubric was developed by a group of Biology Department faculty and has been used by many faculty over the course of multiple years to assess student writing. The student rubrics and weights therein were compared to the departmental rubric weights using one‐sample t‐tests. 15 student papers were chosen randomly using a random number generator. These papers were scored independently using the student rubric and the departmental rubric by the two co‐authors who were not the course instructors. The two scores for each paper were compared using a paired t‐test and a Kolgomorov‐Smirnov test to evaluate for any significant difference in final grade distributions.

## 3 RESULTS

### 3.1 Comparison of weights given to different elements of writing by students and a faculty created departmental rubric

**FIGURE 1:**
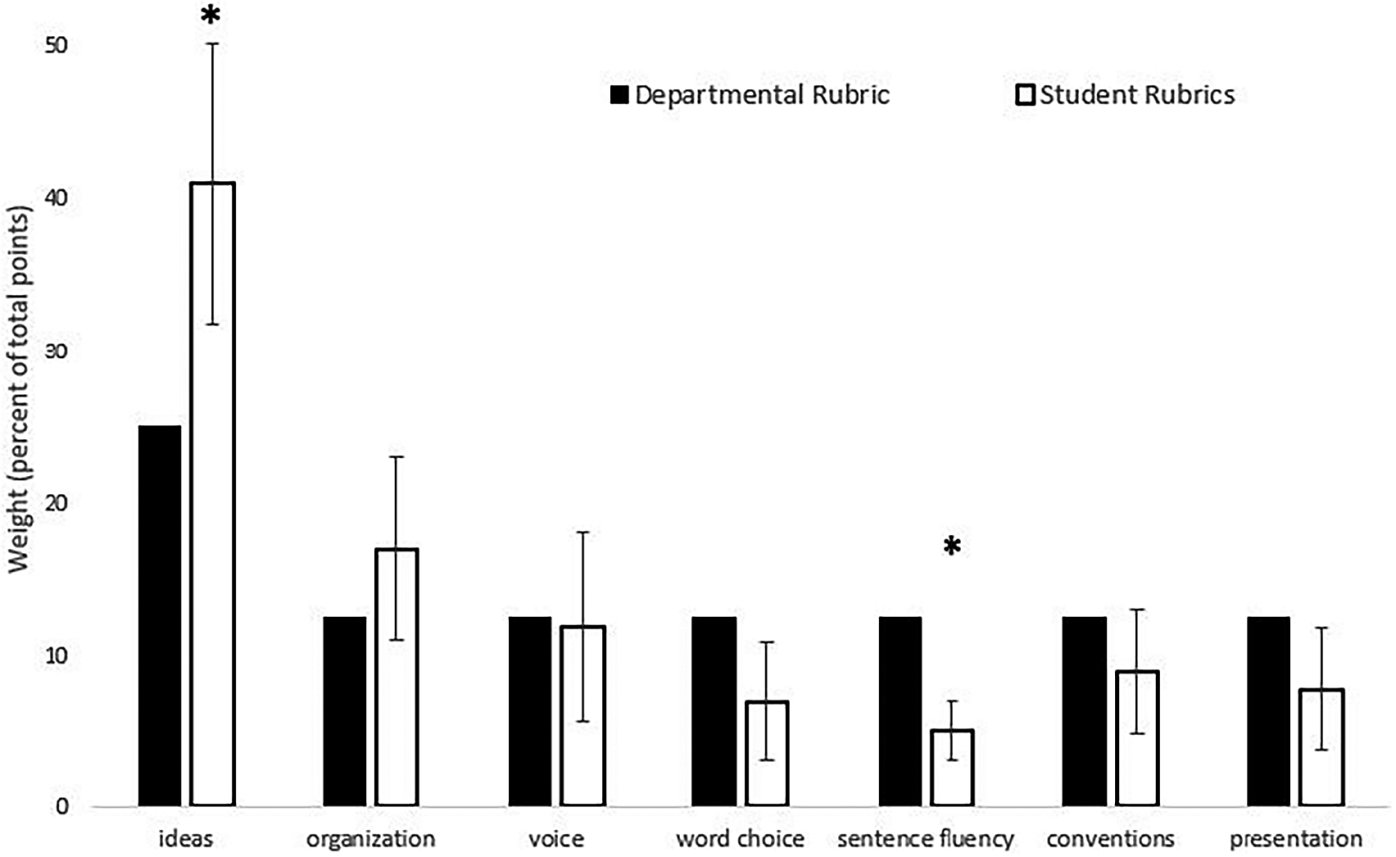
The percentage of total points (weight) assigned to each element of writing by groups of students (white bars) *n* = 10 rubrics, and the Biology Department rubric (black bars) used for assessing SW.

An analysis of ten student‐generated rubrics revealed significant differences between the percentage of total points (weights) assigned to the seven elements of SW as compared to the departmental rubric for assessing SW. Ideas were given significantly higher weights (*p* = .02), and sentence fluency significantly lower weight, compared to the faculty‐based rubric (*p* = .018) (Figure 1). It should be noted, however, that the sample size is relatively small, with only 10 rubrics, meaning that this analysis could be underpowered. It is possible that if more data were added, then more significant differences would be found. As demonstrated by the standard deviations shown in Figure 1, there was substantial disagreement between different groups of students in the weights being assigned to each element, though they overall gave more weight to purely content‐related elements (ideas and organization) than to strictly mechanics‐based elements (conventions and presentations).

Additionally, there is an interesting trend within the student rubric data whereby the students gave vastly unequal weight to the respective elements. For example, there was one group that assigned 70% of the points to “ideas.” Several groups gave no weight to “presentation” or “voice.” The standard deviation between the weights across elements for the departmental rubric was only 5.0, as this rubric gave relatively equal weights to all elements. In the student rubrics, however, the average standard deviation between element weights was 15.5, over 3‐fold higher than the departmental rubric.

### 3.2 Comparison of scores assessed using both the departmental rubric and the student rubrics

**FIGURE 2:**
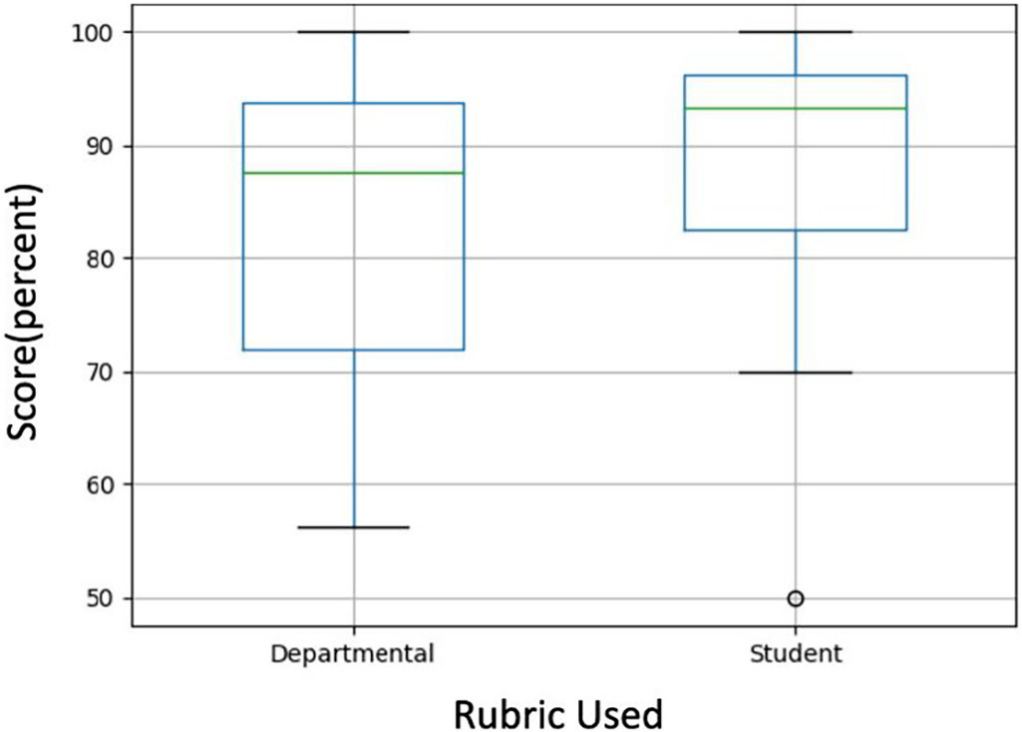
Scores (percent) assessed by two independent investigators using student‐generated rubrics and a faculty‐created departmental writing rubric. Mean ± SD for the student rubric scores and departmental rubric scores were 87.2 ± 0.14 for the student rubrics and 82.5 ± 0.16 respectively. There was no significant difference between the two groups (*p* = .105).

From a random sample of 15 papers, there was no significant difference between scores assessed using the student‐generated rubrics as compared to a faculty designed departmental rubric, according to our paired *t*‐test (*p* = .105). On average, the scores from the student rubrics were higher than the departmentally assessed scores, with a mean of 87.2 ± 0.14 for the student rubrics and 82.5 ± 0.16 for the departmental rubric (Figure 2), though this difference is not statistically distinguishable from zero. According to the grading scale for the course, this is the difference between a B and a B+ letter grade. Moreover, using a Kolgomorov‐Smirnoff test, we are unable to reject the null hypothesis that the two grade distributions are the same. In particular, there is no evidence that the grades from the faculty rubrics are systematically lower (*p* = .549), that the grades from the faculty rubrics are systematically larger (*p* = .936), or that the grade distributions are statistically different (*p* = .925).

## 4 DISCUSSION

This study demonstrates the feasibility of involving students in the assessment process through the creation of student‐generated, collaborative rubrics. The exercise was overall well‐received by students. The instructor anecdotally noted that the students took the workshop seriously and worked to reflect deeply on what constitutes good writing. Overall the instructor found the rubric‐writing workshop to be a positive experience and will continue to implement it in the future.

No significant difference between scores was observed when the same set of 15 papers was scored using both the student‐generated rubrics and a faculty‐generated departmental writing rubric. While the scores were, on average, slightly higher with the student rubrics, the difference amounts to only about one‐half letter grade difference (B to B+) and it is not statistically significant. This amount of potential grade inflation is outweighed by pedagogical gains, such as increased inclusion, discussions about writing, and students gaining autonomy over their work.

This study also provides insight into what students find to be important elements in SW. Previous studies on scholarly writing in higher education across disciplines have found that students often focus on conventions and skills based on learning from secondary education, putting great value on conventions such as correct grammar and spelling, avoiding the first person, and ensuring proper formatting ^2^, ^6^, ^7^. Our results indicate, however, that students place a larger emphasis on ideas and organization at the expense of conventions and presentation. This could be due to differences in student populations, or it could be that these rubrics were used to score an actual summative assessment, causing students to assess their opinions more critically about SW. These results may be different in a course where the content is more writing‐based, such as a composition course, as opposed to the content‐heavy pharmacology course in which this study was completed, making these insights particularly relevant to STEM students.

The students’ emphasis on ideas and content over conventions could be indicative of the inequities that are inherent in SW. It is not a new concept that scholarly writing favors standard English and often excludes or marginalizes by factors of culture and race.^10^, ^11^ This leads to inequities in the assessment of student writing. Part of the motivation behind allowing students to participate in their assessment is to ensure that all voices are heard, particularly from those in marginalized groups. In favoring ideas over mechanics, students could be seeking a way of overcoming these structural inequalities by asking for their ideas and mastery of content to be weighed higher than the ability to adhere to mechanics and the norms of scholarly writing. Given the urgent need to remove barriers and improve retention of underrepresented minority students in pharmacology, further consideration of how we teach and evaluate SW in pharmacology courses is called for.

Baniceru (2016) asserts that students desire consistency in grading across assignments and courses, and clear guidelines and expectations for written assignments. Interestingly, our results show great inconsistency between the point‐value weights assigned to the different elements of SW between groups of students. This could be reflective of the inconsistencies perceived by students between the various disciplines in which they encounter SW, and even between instructors within the same discipline. Additionally, as compared to the standard rubric for the Biology Department, students assigned a vastly unequal weight to the respective elements. For example, there was one group that assigned 70% of the points to “ideas.” Several groups gave no weight to “presentation” or “voice.” This may be indicative of students lacking a holistic view of writing. It is important for instructors to emphasize the importance of all elements of writing to students when assigning writing assignments.

The implementation of writing‐specific pedagogy techniques in an undergraduate pharmacology course is also useful in integrating pharmacology into the University Core Curriculum. The weekly structured writing assignments, rubric workshop, and scaffolded final writing project qualified this “Foundations of Pharmacology” course as a Writing Flag within the University Core Curriculum. Further, engaging students in the topic of social pharmacology (along with other elements throughout the semester) qualified the course as a “Diversity Flag,” again giving students core curriculum credit for this pharmacology class. Pharmacology is inherently an interdisciplinary field, so broadening the scope to fit within the core curriculum does not compromise the pharmacology‐specific content. Adding these curricular incentives increases enrollment and widens the appeal for undergraduate students, attracting a more diverse and talented student body into the field of pharmacology.

Finally, and perhaps the most concerning finding regarding student perceptions of SW in the literature, is the belief that high school writing courses and assignments did not adequately prepare students for the challenges of higher education.^2^, ^12^ This has left students feeling both frustrated and ill‐prepared. Many argue that these negative emotions surrounding SW impede further growth and success.^13^, ^14^ We hope that by involving students in the writing process to a greater degree and by giving them some control over their assessment, students will become more engaged and develop positive feelings about scholarly writing, and improve their scientific writing appreciation and skills.

## AUTHOR CONTRIBUTIONS

All authors contributed to the conceptualization, study design, data collection, data analysis, and writing of the manuscript. All authors approved the final version of the manuscript.

## FUNDING INFORMATION

No funding was associated with this research.

### OPEN RESEARCH BADGES

This article has earned an Open Data badge for making publicly available the digitally‐shareable data necessary to reproduce the reported results. The data is available at https://www.exhibit.xavier.edu/biology_faculty/108/.

## Data Availability

All data associated with this research can be found in the Xavier Exhibit repository at https://www.exhibit.xavier.edu/biology_faculty/108/
